# A Two-Dimensional Manipulation Method for a Magnetic Microrobot with a Large Region of Interest Using a Triad of Electromagnetic Coils

**DOI:** 10.3390/mi13030416

**Published:** 2022-03-07

**Authors:** Hakjoon Lee, Dongjun Lee, Seungmun Jeon

**Affiliations:** Department of Mechanical and Automotive Engineering, Kongju National University, Cheonan 31080, Korea; hacjoon272@naver.com (H.L.); zinith7@naver.com (D.L.)

**Keywords:** magnetic robot, magnetic navigation system, closed-loop control, region of interest

## Abstract

This paper proposes an effective method to manipulate the 2D motions of a magnetic small-scale robot (microrobot) within a relatively large working area using a triad of electromagnetic coils (TEC). The TEC is a combination of three identical circular coils placed at the vertices of an equilateral triangle. Since it is geometrically compact and requires only three control variables (input currents), the TEC can be effectively used to generate various magnetic fields that can be used to maneuver various functional microrobots. In this paper, we established several equations to calculate the input currents of the TEC required to move a microrobot along a designated pathway effectively and precisely. We also constructed an experimental setup to demonstrate and validate the controlled motions of the microrobot using the proposed method. The results showed that the proposed method can effectively improve the TEC’s practical working area (region of interest) for manipulating the microrobot, which can possibly be applied to biomedical and biological applications, including minimally invasive surgery, targeted drug and cargo delivery, microfluidic control, etc.

## 1. Introduction

Untethered small-scale robots with a dimension of a few millimeters or less (microrobots) have gained importance as precise and versatile devices in a variety of applications [1,2,3,4,5]. With the advantages of their miniaturized size and wireless manipulation ability, microrobots can effectively perform various tasks in limited environments where conventional macro-scale robots could not operate as well [6,7]. Magnetic microrobots actuated by a magnetic navigation system (MNS) have especially drawn a lot of attention for biomedical and biological applications, such as minimally invasive surgery [8,9,10], targeted drug and cargo delivery [11,12,13], and microfluidic control [14,15]. Unlike other microrobots, such as the ones based on chemical, ultrasound, or biohybrid mechanisms [16,17], the magnetic microrobot’s principle of manipulation is based on an external magnetic field whose generation, elimination, and modulation can be effectively controlled via the control of several input currents of an MNS [18]. Thus, magnetic microrobots can be simplified and miniaturized with safe and wireless maneuverability, and they can be applied to many different environments including viscous fluid, vacuum space, and living organisms.

Different types of MNSs have been investigated to manipulate various kinds of two-dimensional (2D) or three-dimensional (3D) microrobots precisely and effectively [19,20,21,22]. Jeon et al. proposed a saddle coil system that can be effectively used to manipulate the 2D or 3D motions of a microrobot within a compatible structure for the human body [23]. Kummer et al. proposed an MNS composed of eight electromagnets and a method that can generate 3D motions of a magnetic robot in a specific working space [24]. Nam et al. proposed an MNS utilizing multiple magnetic cores and a closed magnetic circuit to maximize the magnetic field so that the MNS can apply a relatively strong actuation force for various magnetic devices [25].

**Figure 1:**
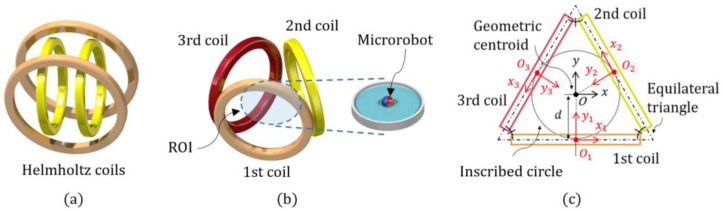
(**a**) Schematic view of the conventional two pairs of Helmholtz coils; (**b**) the TEC; (**c**) the geometrical properties shown in the *xy*-plane.

Although various biomedical and biological procedures take place in a 2D environment, an MNS that is specialized for the 2D manipulation of microrobots has not been investigated well [19,20,21,22,23,24,25]. Since an MNS requires multiple electromagnetic coils with massive turns of wires and an integrated interface to simultaneously regulate the coil currents, the MNS becomes structurally large and electrically inefficient as the microrobot’s allowable working area and degree-of-freedom in motion increases. This can make the construction and operation of the MNS more costly and complex. For instance, conventional MNSs, such as the two pairs of Helmholtz coils shown in Figure 1a, required at least four coils to manipulate the 2D microrobots. Also, each pair of the Helmholtz coils should be different in diameter due to the geometric constraints.

In our previous research, we proposed an MNS simply composed of three identical, circular electromagnetic coils (triad of electromagnetic coils; TEC), as shown in Figure 1b, that can manipulate a microrobot in 2D environments [26]. With a minimal number of electromagnetic coils to manipulate a 2D microrobot, the TEC minimizes or reduces the control effort, energy consumption, and heat dissipation problems using a structurally symmetric and compact system. However, the TEC’s region of interest (ROI) where the microrobot can be properly manipulated within the system was limited to a relatively small central area. This was because the conventional manipulation method of the TEC was based on an assumption that the magnetic field of the TEC is linearly distributed throughout the system with respect to the center. Although this assumption can make it easy to calculate the TEC’s input currents needed to generate a specific motion of the microrobot, it does not take into account the nonlinearly distributed magnetic field of the TEC throughout the system. Thus, the microrobot may deviate from the desired pathway as it moves away from the TEC’s centroid, which can degrade the accuracy of the microrobot’s mechanical motion as it performs functional motions actuated by the TEC.

In this paper, we propose an effective method that enables the TEC to manipulate a microrobot within a relatively large area of the system effectively and precisely. We established mathematical equations to precisely calculate the TEC’s magnetic field and the corresponding magnetic force of the microrobot at an arbitrary position in the TEC by using local coordinates and vector transformations. We constructed constraint equations to effectively maneuver the microrobot’s 2D motions with respect to the different positions of the microrobot. We also applied a closed-loop controller to the method to make the microrobot move along a predetermined programmed pathway in a real-time manner. We then constructed an experimental setup and demonstrated several controlled 2D motions of a microrobot to verify the proposed method.

## 2. Manipulation of the 2D Motions of a Microrobot Using the TEC

### 2.1. Principle of Manipulation

The magnetic torque and force exerted on a microrobot in a magnetic field can be expressed by the following respective equations:

(1) T=m×B

(2) $$F=\nabla \left(m\cdot B\right)=\frac{\partial B}{\partial X}m$$

where m, B, and $\frac{\partial B}{\partial X}$ are the magnetic moment of the microrobot, the external magnetic field, and the gradient matrix of B with respect to a vector (X) representing the coordinate system, respectively.

The magnetic field of the *k*-th coil at an arbitrary position ($x_{k}$) with respect to the center of the coil ($O_{k}$), as shown in Figure 1c, can be analytically calculated using the Biot−Savart’s law. For the convenience of calculating the transformation and rotation of the magnetic field, the magnetic field is expressed with Cartesian coordinates, as follows [27]: (substitutions are used for simplicity, $\rho_{k}{}^{2}\equiv x_{k}{}^{2}+z_{k}{}^{2}$,$\;r_{k}{}^{2}\equiv x_{k}{}^{2}+y_{k}{}^{2}+z_{k}{}^{2}$, $\alpha_{k}{}^{2}\equiv a_{k}{}^{2}+r_{k}{}^{2}-2a_{k}\rho_{k}$, $\beta_{k}{}^{2}\equiv a_{k}{}^{2}+r_{k}{}^{2}+2a_{k}\rho_{k}$, and $k_{k}{}^{2}\equiv 1-\alpha_{k}{}^{2}/\beta_{k}{}^{2}$):

(3) $$B_{k}^{O_{k}}\left(x_{k}\right)=\frac{\mu_{0}i_{k}}{2\pi \alpha_{k}{}^{2}\beta_{k}}\left[\begin{array}{c} \frac{x_{k}y_{k}}{\rho_{k}{}^{2}}\left(\left(\alpha_{k}{}^{2}+r_{k}{}^{2}\right)E\left(k_{k}{}^{2}\right)-\alpha_{k}{}^{2}K\left(k_{k}{}^{2}\right)\right)\\ \left(\alpha_{k}{}^{2}+r_{k}{}^{2}\right)E\left(k_{k}{}^{2}\right)-\alpha_{k}{}^{2}K\left(k_{k}{}^{2}\right)\\ \frac{y_{k}z_{k}}{\rho_{k}{}^{2}}\left(\left(\alpha_{k}{}^{2}-r_{k}{}^{2}\right)E\left(k_{k}{}^{2}\right)+\alpha_{k}{}^{2}K\left(k_{k}{}^{2}\right)\right) \end{array}\right]$$

where $x_{k}$, $y_{k}$, $z_{k}$, $a_{k}$, $i_{k}$, $\mu_{0}$, K, and E are the *x*-, *y*-, and *z*-axial positions, the radius and current of the *k*-th coil, the permeability of free space, and the complete elliptic integral of the first and second kinds, respectively. The TEC is a combination of three identical circular coils placed at the vertices of an equilateral triangle, as shown in Figure 1. The overall magnetic field of the TEC can, thus, be calculated by the superposition of each coil’s magnetic field. By using the local coordinates and the vector transformations, the TEC’s magnetic field and its gradient matrix at an arbitrary position, x, in the *xy*-plane with respect to the centroid of the TEC, can be expressed as the following simple equations:

(4) $$B_{\mathrm{TEC}}=\overset{3}{\underset{k=1}{\sum }}R_{z}^{\theta_{k}}B_{k}^{O_{k}}\left(x_{k}\right)=P_{B}\left(x\right)i_{\mathrm{TEC}}$$

(5) $$\frac{\partial B_{\mathrm{TEC}}}{\partial X}=\sum_{k=1}^{3}R_{z}^{\theta_{k}}\frac{\partial B_{k}^{O_{k}}\left(x_{k}\right)}{\partial X_{k}}R_{z}^{-\theta_{k}}=J_{B}\left(x,\;i_{\mathrm{TEC}}\right)$$

where $X_{k}$ $R_{z}^{\theta_{k}}$, $P_{B}$, $J_{B}$, and $i_{\mathrm{TEC}}$ are the vector representing the coordinate system with respect to the *k*-th coil’s origins ($O_{k}$), the *z*-directional rotation matrix with a rotation angle of $\theta_{k}$, the coefficient and Jacobian matrices of the TEC’s magnetic field, and ${\left[i_{1}\;i_{2}\;i_{3}\right]}^{T}$, respectively. Also, from the geometry of the TEC’s equilateral triangle shown in Figure 1b, Equations (4) and (5) satisfy the following equations:

(6) $$\theta_{k}=\frac{2\pi \left(k-1\right)}{3}$$

(7) $$x_{k}=R_{z}^{-\theta_{k}}\left(x+dR_{z}^{\theta_{k}}j\right)$$

where *d* and j are the radius of the TEC’s inscribed circle and a *y*-directional unit vector of X, respectively. Assuming the microrobot has a planar fluidic working environment, such as the one shown in Figure 1, the microrobot’s rotational motion is relatively unimpeded compared to its translational motion. Thus, it can be assumed that the microrobot always matches (or follows) the applied magnetic field ($m∥B_{\mathrm{TEC}}$) once the magnetic field changes slower than the critical step-out speed of the microrobot [28]. At speeds in excess of this threshold, the microrobot may not be able to follow the magnetic field due to the increased inertia effect and shows unpredictable rattling motions. Therefore, the orientation of the microrobot (the direction of m) can be expressed in terms of the TEC’s magnetic field ($B_{\mathrm{TEC}}$), and the magnetic force of the microrobot can, thus, be simplified as the following equation:

(8) $$F_{\mathrm{TEC}}=J_{B}\left(x,\;i_{\mathrm{TEC}}\right)m=P_{F}\left(x\right)i_{\mathrm{TEC}}$$

where $P_{F}$ is the integrated coefficient matrix of the TEC’s magnetic field and the microrobot.

### 2.2. Generating 2D Magnetic Force of the Microrobot

Considering that each coil axis of the TEC and the microrobot’s magnetic moment always lie in the *xy*-plane, it can be assumed that both the *z*-directional components of $B_{\mathrm{TEC}}$ and $F_{\mathrm{TEC}}$ always equal zero during the microrobot’s 2D motions. Therefore, a constraint equation for the 2D aligning and propelling motions of the microrobot in the *xy*-plane can be expressed as follows:

(9) $$\left[\begin{array}{c} B_{\mathrm{TEC}}^{xy}\\ F_{\mathrm{TEC}}^{xy} \end{array}\right]=\left[\begin{array}{c} P_{B}^{xy}\left(x\right)\\ P_{F}^{xy}\left(x\right) \end{array}\right]i_{\mathrm{TEC}}=\left[\begin{array}{c} B_{0}\sin\alpha_{B}\\ B_{0}\cos\alpha_{B}\\ F_{0}\cos\alpha_{F}\\ F_{0}\sin\alpha_{F} \end{array}\right]$$

where $B_{0}$, $\alpha_{B}$, $F_{0}$, $\alpha_{F}$, $B_{\mathrm{TEC}}^{xy}$, $F_{\mathrm{TEC}}^{xy}$, $P_{B}^{xy}$, and $P_{F}^{xy}$ are the desired magnitude and direction of the magnetic field and force exerted on the microrobot and the column vectors composed of the *x* and *y*-directional components of $P_{B}$ and $P_{F}$, respectively. However, considering that the dimension of the coefficient matrix is 4-by-3, Equation (9) is an overdetermined equation whose solution for $i_{\mathrm{TEC}}$ only exists in limited conditions. In other words, four variables of the microrobot’s 2D mechanical motion ($F_{0}$, $B_{0}$, $\alpha_{F}$, and $\alpha_{B}$) cannot be independently varied by the three input currents of the TEC. Thus, one should examine the existence of a solution of Equation (9) using complex linear algebraic computations, including the derivations of the singular value decomposition and the pseudoinverse matrix according to different values of x, $B_{\mathrm{TEC}}^{xy}$, and $F_{\mathrm{TEC}}^{xy}$ at every instance of the microrobot’s motions.

Meanwhile, for certain types of microrobots with 2D symmetric geometries, such as disk-, ring-, and sphere-type microrobots, their mechanically apparent motions can be characterized by positional variables ($F_{0}$ and $\alpha_{F}$) rather than directional variables ($B_{0}$ and $\alpha_{B}$). This becomes more effective as the microrobot gets smaller because of the viscosity effect in a low Reynold number flow [29]. In this research, it is assumed that only the magnitude ($F_{0}$) and direction ($\alpha_{F}$) of the magnetic force are the independent variables for the 2D mechanical motions of the microrobot. In this case, Equation (9) can be reduced to a simplified one, as follows:

(10) $$F_{\mathrm{TEC}}^{xy}=P_{F}^{xy}\left(x\right)i_{\mathrm{TEC}}=\left[\begin{array}{c} F_{0}\cos\alpha_{F}\\ F_{0}\sin\alpha_{F} \end{array}\right]$$

However, Equation (10) is rather an underdetermined equation that can yield numerous solutions. To obtain a unique and useful solution ($i_{\mathrm{TEC}}$) for a given condition of the microrobot motion ($F_{0}$ and $\alpha_{F}$), we applied another condition to Equation (10) that the microrobot always aligns along a direction in which the TEC can consume the minimum electric power while generating the required magnetic force of the microrobot, as follows:

(11) $$\alpha_{B}=\underset{\alpha_{B}}{\min}\|i_{\mathrm{TEC}}\|$$

Therefore, one can calculate the three input currents of the TEC required to manipulate the microrobot motions and can minimize the electric power consumption of the TEC at the same time.

In this study, we also employed a controller for the manipulation method to make the microrobot move along a programmed pathway in a closed-loop manner. For a given pathway of the microrobot in the *xy*-plane, the position error of the microrobot can be expressed as follows:

(12) $$e\left(t\right)=x_{\mathrm{ref}}\left(t\right)-x_{\mathrm{obs}}\left(t\right)$$

where $x_{\mathrm{ref}}\left(t\right)$ and $x_{\mathrm{obs}}\left(t\right)$ are the referenced and observed 2D positions of the microrobot with respect to time, *t*, respectively. By using a proportional-integral-derivative (PID) controller, the magnetic force at the instance of time, *t*, required to make the microrobot follow a given pathway can be obtained as follows:

(13) $$F_{\mathrm{TEC}}^{xy}\left(t\right)=-\left(K_{P}\|e\left(t\right)\|+K_{I}\int \|e\left(t\right)\|dt+K_{D}\frac{d\|e\left(t\right)\|}{dt}\right)\frac{e\left(t\right)}{\left\|e\left(t\right)\right\|}$$

where $K_{P}$, $K_{I}$, and $K_{D}$ are the gains in the proportional, integral, and derivative controllers, respectively. The proper values of $K_{P}$, $K_{I}$, and $K_{D}$ to ensure the microrobot continuously follows the pathway, while reducing the position error, the maximum overshoot, and the response time, can be obtained by various heuristic tuning methods [30]. Therefore, one can effectively and precisely manipulate the 2D motions of a microrobot located at an arbitrary position in a plane via the control of only three input currents of the TEC by using Equations (1)–(13).

## 3. Results and Discussion

**Figure 2:**
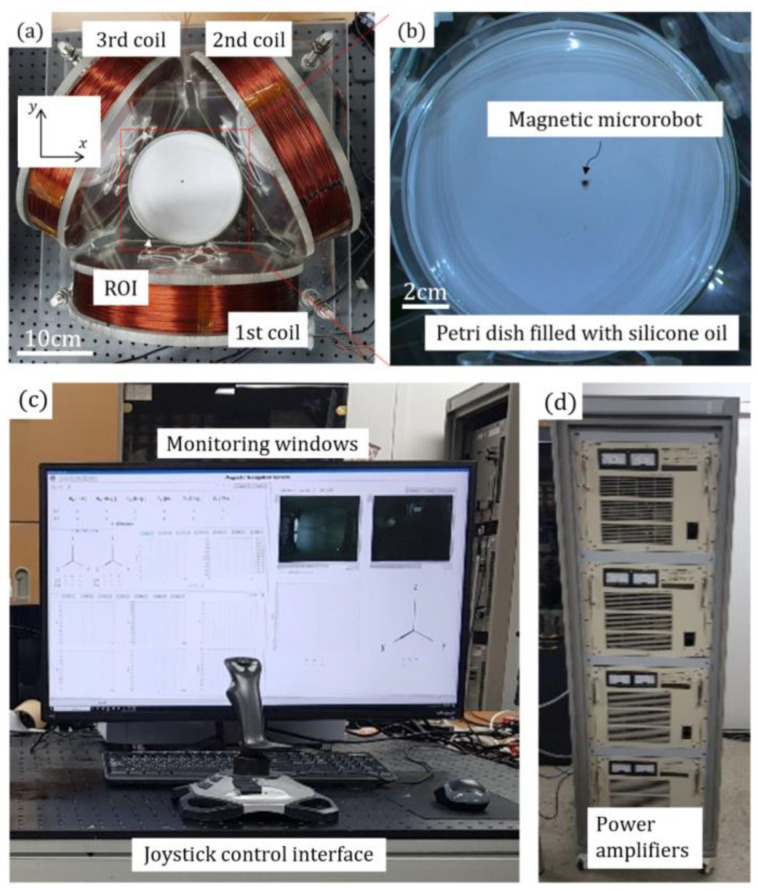
Experimental setup of the (**a**) TEC, (**b**) magnetic microrobot, (**c**) control panel, and (**d**) power amplifiers to demonstrate the 2D microrobot motions using the TEC.

**Figure 3:**
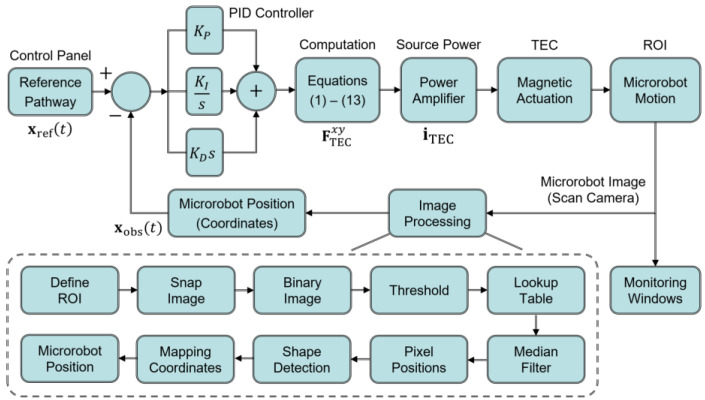
Schematic diagram of the closed-loop control system implemented to the experimental setup shown in Figure 2. The system also includes a real-time image processing procedure using a LabVIEW visual object tracking system. Observed positions of the microrobot may be different from its real positions because of the limitations of the scan camera’s optical resolution and the computational errors during the image processing.

In this research, we conducted several experiments to demonstrate the TEC’s ability to manipulate the microrobot’s 2D motions. We first constructed an experimental setup, as shown in Figure 2. The nominal radius and the number of turns of each circular coil were identically 125 mm and 1300 turns, respectively. A copper wire with a diameter of 1 mm was used for the TEC. Each coil of the TEC was connected to three respective power amplifiers (Precision Power Amplifier 4510, NF Corporation, Yokohama, Japan), which were integrated into a control panel using a LabVIEW hardware interface (PCle-6738, National Instruments, Austin, TX, USA). Equations (1)–(13) were implemented in the system using a LabVIEW graphical programming language. In this way, the magnetic field of the TEC can be precisely generated by simultaneously regulating the TEC’s input currents using the control panel. We also implemented an area scan camera (acA2040-120uc, Basler AG, Ahrensburg, Germany) mounted over the top of the TEC to obtain real-time images of the microrobot. The resolution and maximum frame rate were 2048 pixels × 1536 pixels and 120 frames per second, respectively. Figure 3 shows the overall structure of the constructed control system, including the image processing procedure. We obtained the real-time positions of the microrobot from the acquired images of the microrobot by using a LabVIEW Vision Assistant platform (National Instruments). The overall procedure shown in Figure 3 took approximately 40 milliseconds to execute one loop.

In this research, we assumed that a microrobot is a structure that ranges from several millimeters down to few micrometers in all dimensions. We used a transversely magnetized disk-type neodymium magnet as a microrobot. The diameter, height, and magnetization of the magnet were 3 mm, 1 mm, 955 kA/m, respectively. Considering the resolution and field of view of the scan camera, the microrobot should be sufficiently large so that its movement can be clearly observed by the scan camera within a large ROI of the TEC. Submillimeter-scale microrobots with functional structures can also be manipulated by the system, provided that a microscopic image acquisition device with a higher resolution is used. We then constructed a horizontal petri dish filled with a transparent, viscous silicone oil (100 cP) so that the microrobot motions could be clearly and steadily observed during the operation.

**Figure 4:**
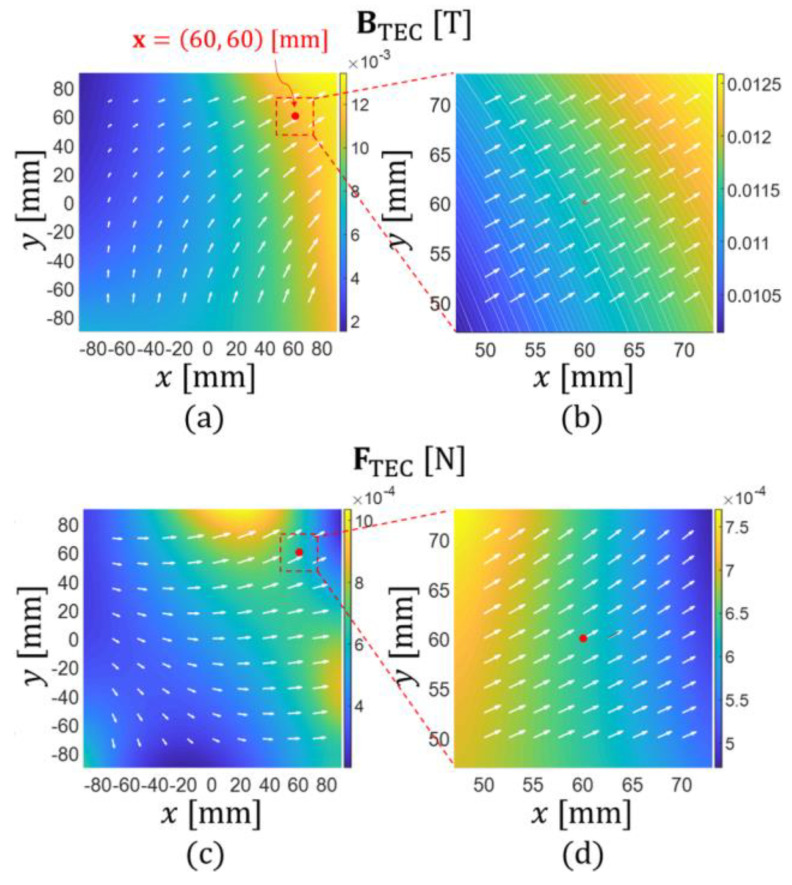
(**a**,**b**) Distribution of the magnetic field inside the TEC; (**c**,**d**) the corresponding magnetic force generated to apply a magnetic force of $\left(F_{0},\alpha_{F}\right)=\left(500\;\mu N,\;30{}^{\circ}\right)$ to the microrobot located at $x={\left[60\;\mathrm{mm},\;60\;\mathrm{mm},0\right]}^{T}$. In this case, the optimal $\alpha_{B}$ is approximately 39°.

Before demonstrating the microrobot motions, we first verified the proposed manipulation method of the microrobot by simulating the magnetic field of the TEC and the corresponding magnetic force acting on the microrobot, as shown in Figure 4. Figure 4a,b shows the distribution of the magnetic field near the central area of the TEC depicted by MATLAB (MathWorks, Inc., Natick, MA, USA) graphic scripts. The TEC’s input currents were calculated using Equations (1)–(11) under the condition that a magnetic force of $\left(F_{0},\alpha_{F}\right)=\left(500\;\mu N,\;30{}^{\circ}\right)$ was to be applied to the microrobot located at $x={\left[60\;\mathrm{mm},\;60\;\mathrm{mm},0\right]}^{T}$. In this magnetic field, the microrobot can move along the direction of $\alpha_{F}=30{}^{\circ}$ while aligning along the direction of $\alpha_{B}=39{}^{\circ}$.

**Figure 5:**
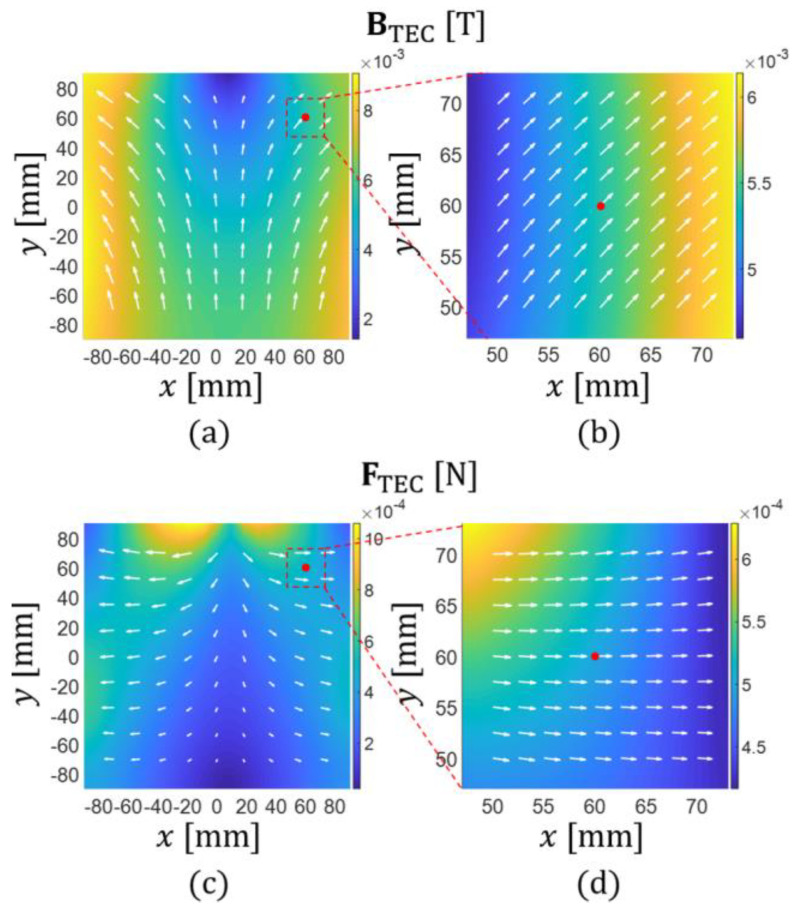
(**a**,**b**) The distribution of the magnetic field inside the TEC; (**c**,**d**) the corresponding magnetic force generated to apply a magnetic force of $\left(F_{0},\alpha_{F}\right)=\left(500\;\mu N,\;0{}^{\circ}\right)$ to the microrobot located at $x={\left[60\;\mathrm{mm},\;60\;\mathrm{mm},0\right]}^{T}$. In this case, the optimal $\alpha_{B}$ is approximately 45°.

**Figure 6:**
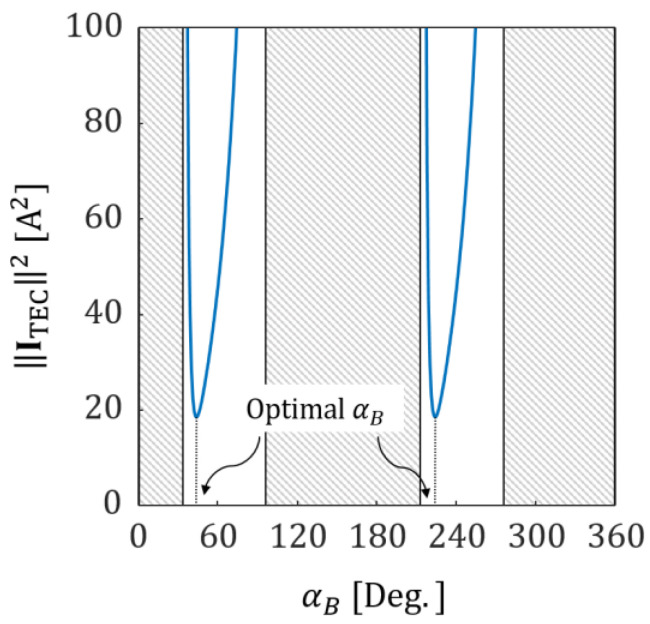
The variation in the TEC’s overall currents with respect to the aligning direction of the microrobot required to generate the magnetic force shown in Figure 5.

Figure 5 shows another case of the simulation. In this case, the TEC’s input currents were calculated under the condition that a magnetic force of $\left(F_{0},\alpha_{F}\right)=\left(500\;\mu N,\;0{}^{\circ}\right)$ was to be applied to the microrobot located at $x={\left[60\;\mathrm{mm},\;60\;\mathrm{mm},0\right]}^{T}$. Figure 6 shows the variation in the TEC’s overall currents (square of norm of the current vector) with respect to the aligning direction of the microrobot ($\alpha_{B}$) required to generate the magnetic force shown in Figure 5. In Figure 6, there are two symmetric optimal values of $\alpha_{B}$ (45° and 225°) derived from the symmetry of the microrobot’s N−S dipole moment. Considering the desired moving direction of the microrobot ($\alpha_{F}=0{}^{\circ}$), $\alpha_{B}=45{}^{\circ}$ whose angle between $\alpha_{F}$ is smaller than the other was chosen for the solution. Thus, the results show that the TEC can generate a magnetic field, precisely and effectively, that can be used to manipulate a microrobot located at an arbitrary position in the TEC.

**Figure 7:**
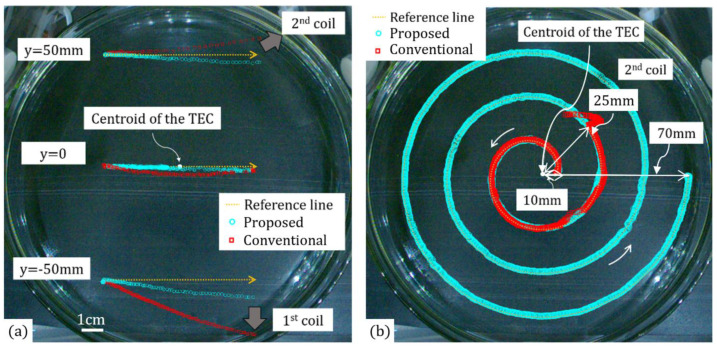
(**a**) Open-loop manipulation trajectories of the microrobot along three straight pathways actuated by the conventional and proposed manipulation methods. (**b**) Closed-loop manipulation trajectories of the microrobot along a spiral pathway actuated by the conventional and proposed manipulation methods. (See Supplemental Videos).

In this research, we also verified the proposed manipulation method by demonstrating several controlled motions of the microrobot. Without the application of the closed-loop control, we first examined the efficacy of the proposed method (Equations (1)–(11)) compared to that of the conventional manipulation method, as shown in Figure 7a. Since the conventional method relies on the assumption that the microrobot is always located at the center (centroid) of the TEC, this method is not applicable to cases in which the microrobot should move within a relatively large area (ROI).

Figure 7a shows the open-loop manipulation trajectories of the microrobot along three straight lines $(\left(F_{0},\alpha_{F}\right)=\left(500\;\mu N,\;0{}^{\circ}\right)$) actuated using the conventional and proposed manipulation methods. For the central straight line, both methods were able to move the microrobot along the reference line with a relatively small position error. However, the microrobot deviated from the desired pathway when it moved along a straight line located away from the center. In this case, the conventional method showed a much larger position error, but the proposed method also showed a significant position error because the open-loop control cannot take into account the changes in position of the microrobot during manipulation. The average speeds of the microrobot along the upper, central, and lower straight lines were measured to be 14.3 mm/s, 8.6 mm/s, and 22.1 mm/s, respectively. Although the same constant magnetic force was used for the manipulation, the microrobot could not show a uniform speed at different locations due to the variating viscous friction effect during motion.

On the other hand, with the application of the closed-loop control, the proposed method (Equations (1)–(13)) was able to move the microrobot along a complex spiral pathway with a much smaller position error, as shown in Figure 7b. In this case, the values of $K_{P}$, $K_{I}$, and $K_{D}$ were adjusted to 20 $\mathrm{kg}/s^{2}$, 0 $\mathrm{kg}/s^{3}$, and 0.002 kg/s, respectively, to make the microrobot move along the pathway with a maximum velocity of 2.50 mm/s. The maximum, average, and standard deviation of the position error were measured to be 2.1 mm, 0.66 mm, and 0.79 mm, respectively. Considering the size of the microrobot compared to the field of view of the scan camera shown in Figure 7b, this error is sufficiently small. It was seen that the microrobot’s position error is affected by both the values of $K_{P}$, $K_{I}$, and $K_{D}$ and the error between the observed and real positions of the microrobot. The position error and the maximum allowable moving speed of the microrobot can further be adjusted by refining the PID controller or by using a microscopic scan camera with a higher image resolution.

The conventional method, however, could only move the microrobot along the same spiral pathway near the center of the TEC, even with the application of the closed-loop control. Although the same values of $K_{P}$, $K_{I}$, and $K_{D}$ were used, the conventional method could only manipulate the microrobot within a relatively small central area of the TEC (25 mm in radius) because of the increased error in calculating the magnetic field and the magnetic force, regardless of the application of the closed-loop control (see Supplemental Videos S1 and S2).

The experimental results verified that the proposed microrobot manipulation method can effectively increase the practical working area of the TEC so that the microrobot can be used to perform various functional motions precisely and effectively.

## 4. Conclusions

In this paper, we proposed an effective method to manipulate the 2D motions of a microrobot within a relatively large working area via a geometrically compact and electrically efficient electromagnetic coil system. Simulated and experimental results verified the proposed method. Although further investigations, such as for the small-scale localization and the higher degree-of-freedom manipulation of a functional microrobot, remain for future work, this research can contribute to the development of structurally and electrically realistic MNSs and the relevant manipulation skills for various small-scale robot applications.

## Data Availability

The data that support the findings of this study are available from the corresponding author upon reasonable request.

## Supplementary Materials

The following supporting information can be downloaded at: https://www.mdpi.com/article/10.3390/mi13030416/s1, Video S1: Open-loop manipulation of the microrobot along straight pathways.; Video S2: Closed-loop manipulation of the microrobot along a spiral pathway.
